# Multi-omics analysis reveals the involvement of origin recognition complex subunit 6 in tumor immune regulation and malignant progression

**DOI:** 10.3389/fimmu.2023.1236806

**Published:** 2023-10-12

**Authors:** Jinfeng Zhu, Qitong Chen, Liyun Zeng, Hongyu Gao, Tong Wu, Yeqing He, Jiachi Xu, Jian Pang, Jing Peng, Yueqiong Deng, Yi Han, Wenjun Yi

**Affiliations:** ^1^ Department of General Surgery, The Second Xiangya Hospital, Central South University, Changsha, Hunan, China; ^2^ Clinical Research Center For Breast Disease In Hunan Province, Changsha, Hunan, China

**Keywords:** *Orc6*, pan-cancer, prognosis, immunotherapy, tumor microenvironment

## Abstract

**Background:**

Origin recognition complex 6 (*ORC6*) is one of the six highly conserved subunit proteins required for DNA replication and is essential for maintaining genome stability during cell division. Recent research shows that *ORC6* regulates the advancement of multiple cancers; however, it remains unclear what regulatory impact it has on the tumor immune microenvironment.

**Methods:**

Unpaired Wilcoxon rank sum and signed rank tests were used to analyze the differences in the expression of *ORC6* in normal tissues and corresponding tumor tissues. Multiple online databases have evaluated the genetic alterations, protein expression and localization, and clinical relevance of *ORC6*. To evaluate the potential prognostic impact and diagnostic significance of *ORC6* expression, we carried out log-rank, univariate Cox regression, and receiver operating characteristic curve analysis. The ICGC-LIRI-JP cohort, CGGA-301 cohort, CGGA-325 cohort, CGGA-693 cohort, and GSE13041 cohort were used for external validation of the study findings. The associations between *ORC6* expression and immune cell infiltration, immune checkpoint expression, and immunotherapy cohorts was further analyzed. To explore the functional and signaling pathways related to *ORC6* expression, gene set enrichment analysis was performed. To clarify the expression and function of *ORC6* in hepatocellular carcinoma (LIHC) and glioma, we conducted *in vitro* experiments.

**Results:**

Expression of *ORC6* is upregulated in the majority of cancer types and is associated with poor patient prognosis, notably in cases of LIHC and gliomas. In addition, *ORC6* may be involved in multiple signaling pathways related to cancer progression and immune regulation. High expression of *ORC6* correlates with an immunosuppressive state in the tumor microenvironment. The results of further immunotherapy cohort analysis suggested that patients in the *ORC6* high-expression group benefited from immunotherapy. Inhibiting *ORC6* expression suppressed the proliferative and migratory abilities of LIHC and glioma cells.

**Conclusion:**

High expression of *ORC6* may be used as a biomarker to predict the poor prognosis of most tumor patients. The high expression of *ORC6* may be involved in the regulation of the tumor immunosuppressive environment, and it is expected to become a molecular target for inhibiting tumor progression.

## 1 Introduction

Worldwide, cancer presents a life-threatening situation and is one of the most economically burdensome diseases (1). Currently, no treatment for cancer is absolutely effective. As research advances, scientists are increasingly concentrating on the shared characteristics of different malignant tumors to uncover their underlying causes and create targeted inhibitors for cancer therapy (2). For instance, PD-L1 levels are often increased in different cancer types, and recent studies indicate that many oncogenic signaling pathways lead to this overexpression. Antagonistic antibodies against the inhibitory immune checkpoint receptor *PD-1* or its ligand *PD-L1* have shown promise in the treatment of various cancers, leading to significant improvement in patient survival rates (3). Protein tyrosine kinases from the human epidermal growth factor receptor family, such as *EGFR* and *HER2*, are important therapeutic targets for many malignancies, including non-small cell lung cancer, breast cancer, and gastroesophageal cancer, particularly colorectal cancer (4). Aldehyde dehydrogenase (ALDH) serves as a cancer stem cell biomarker across various cancers. Clinically, ALDHs are also regarded as indicators of poor prognosis in solid cancers. Targeting ALDHs may impede cancer stem cells in solid tumors, thereby achieving therapeutic effects (5). Therefore, analyzing the differential expression of genes across cancers, screening valuable genes, and exploring their correlation with clinical prognosis and the tumor immune microenvironment will promote the further development of tumor-targeted therapy and immunotherapy.

The origin recognition complex (ORC) is a vital six-subunit protein that is highly conserved across species and plays a crucial role in DNA replication. It is essential for maintaining genome stability during cell division (6). *ORC6*, the smallest subunit of human ORC, is primarily involved in chromosome segregation, DNA replication, and cell division. It localizes to replication forks to carry out these functions (7). *ORC6* is a cofactor in the mismatch repair (MMR) complex that promotes efficient mismatch repair (8). In recent years, research has identified a correlation between elevated *ORC6* expression and adverse prognostic outcomes in patients with colorectal cancer (9), renal clear cell carcinoma (10), gastric adenocarcinoma (11), and breast cancer (12). Current research on *ORC6* in tumors is restricted to specific types of human cancers. There has been no systematic multi-omics analysis across different types of cancer, notably in liver hepatocellular carcinoma (LIHC) and glioma (GBMLGG).

The research found that *ORC6* was frequently overexpressed in various cancer types and was associated with adverse survival outcomes. Additionally, the biological function of *ORC6* may be linked to RNA modifications, DNA methylation, and the tumor immune microenvironment. By examining *ORC6* across cancers, we observed that it significantly contributes to the development of LIHC and GBMLGG. Our study reveals that *ORC6* acts as an independent risk factor for the overall prognosis of LIHC and GBMLGG. Subsequently, we conducted *in vitro* experiments to elucidate whether *ORC6* promotes the progression of LIHC and GBMLGG. Together, our investigation provides a comprehensive understanding of the tumorigenic role of *ORC6* in different cancers and indicates that *ORC6* could be a dependable biomarker for predicting the clinical prognoses and immune landscapes in patients with LIHC and GBMLGG.

## 2 Materials and methods

### 2.1 Data preprocessing and differential expression analysis

We obtained a unified and standardized pan-cancer dataset (TCGA TARGET GTEx, https://xenabrowser.net/) from the UCSC database. Furthermore, we extracted the *ORC6* gene values from each sample and applied log_2_ (x+1) transformation for each value. The Sangerbox (13) online tool was used for visualization. In addition, we acquired validation cohorts from external sources, including the International Cancer Genome Consortium (ICGC), Chinese Glioma Genome Atlas Project (CGGA), and Gene Expression Omnibus (GEO) databases. These cohorts included the ICGC-LIRI-JP cohort, CGGA-301 cohort, CGGA-325 cohort, CGGA-693 cohort, and GSE13041 cohort. Cancer-type abbreviations are listed in **Table 1**.

**Table 1:** Tumor types and abbreviations.

Abbreviation	Full name
ACC	Adrenocortical carcinoma
ALL	Acute Lymphoblastic Leukemia
AML	Acute myeloid leukemia
AST	Astrocytoma
BLCA	Bladder Urothelial Carcinoma
BRCA	Breast invasive carcinoma
CESC	Cervical squamous cell carcinoma and endocervical adenocarcinoma
CHOL	Cholangiocarcinoma
CML	Chronic myelogenous leukemia
COAD	Colon adenocarcinoma
COADREAD	Colon adenocarcinoma/Rectum adenocarcinoma
DLBC	Lymphoid Neoplasm Diffuse Large B-cell Lymphoma
ESCA	Esophageal carcinoma
GBM	Glioblastoma multiforme
GBMLGG	Glioma
HGG	High-grade glioma
HNSC	Head and Neck squamous cell carcinoma
KICH	Kidney Chromophobe
KIPAN	Pan-kidney cohort (KICH+KIRC+KIRP)
KIRC	Kidney renal clear cell carcinoma
KIRP	Kidney renal papillary cell carcinoma
LAML	Acute Myeloid Leukemia
LGG	Brain Lower Grade Glioma
LIHC	Liver hepatocellular carcinoma
LSCC	Lung squamous cell cancer
LUAD	Lung adenocarcinoma
LUSC	Lung squamous cell carcinoma
MEL	Melanoma
MESO	Mesothelioma
NSCLC	Non-small cell lung cancer
ODG	Oligodendroglioma
OV	Ovarian serous cystadenocarcinoma
PAAD	Pancreatic adenocarcinoma
PCPG	Pheochromocytoma and Paraganglioma
PRAD	Prostate adenocarcinoma
RB	Retinoblastoma
RCC	Renal cell carcinoma
READ	Rectum adenocarcinoma
SARC	Sarcoma
SKCM	Skin Cutaneous Melanoma
STAD	Stomach adenocarcinoma
STES	Stomach and Esophageal carcinoma
TGCT	Testicular Germ Cell Tumors
THCA	Thyroid carcinoma
THYM	Thymoma
UCEC	Uterine Corpus Endometrial Carcinoma
UCS	Uterine Carcinosarcoma
UM	Uveal Melanoma
UVM	Uveal Melanoma
WT	High-Risk Wilms Tumo

### 2.2 Genetic alterations, localization, and interaction network of *ORC6*

The gene mutation type and frequency of *ORC6* in the TCGA pan-cancer dataset were explored by accessing cBioPortal (https://www.cbioportal.org/). We obtained the copy number variation (CNV) dataset at gene level 4 from all TCGA samples processed by GISTIC software (14) through GDC (https://portal.gdc.cancer.gov/). We used the unpaired Wilcoxon rank sum test or the Wilcoxon signed rank test to compare the values between the two groups and the Kruskal–Wallis test for differences among multiple groups.

The Human Protein Atlas (HPA, https://www.proteinatlas.org/) was utilized to obtain images of the subcellular localization of *ORC6* protein in cancer cells (HEK293 and PC-3) by immunofluorescence staining of cells. Furthermore, the subcellular localization of the *ORC6* gene was obtained through the Genecards database.

The comPPI website (http://comppi.linkgroup.hu/) was utilized to analyze the protein-protein interaction network of *ORC6*. The minimum interaction score was 1, and the edge width was scaled based on the interaction score.

### 2.3 The relationship among *ORC6* expression levels, clinical characteristics, and prognosis

The correlation of *ORC6* expression with clinical features was assessed by Spearman correlation analysis. We performed univariate Cox regression analysis to investigate the prognostic significance of *ORC6* expression in predicting the disease-free interval (DFI), progression-free interval (PFI), overall survival (OS), and disease-specific survival (DSS) in pan-cancer cohorts. We then utilized forest plots for a graphical representation of these results.

TCGA data were curated to extract *ORC6* expression levels in transcripts per million (TPM) format, followed by data normalization using log2(TPM+1). Survival data of matched samples were integrated and subsequently subjected to optimal grouping truncation using the ‘surv_cutpoint’ function from the ‘survminer’ package. The aim was to distinguish between the high and low *ORC6* expression groups. Prognostic differences between the high- and low-expression groups were evaluated using the log-rank test. RNA-seq data in TPM format from TCGA and GTEx were uniformly processed through the Toil pipeline, as sourced from UCSC XENA (https://xenabrowser.net/datapages/). *ORC6* expression levels corresponding to TCGA cancer samples and GTEx normal tissue samples for each cancer type were extracted. The data were normalized using Log2(TPM+1). To assess the diagnostic accuracy for tumor detection, we employed the ‘pROC’ package to calculate sensitivity and specificity. Diagnostic value was quantified by the area under the curve (AUC), with a value of 1.0 indicating perfect diagnostics and 0.5 representing no diagnostic value. An AUC greater than 0.85 was considered to possess a high diagnostic value.

### 2.4 Correlation of *ORC6* expression with DNA methylation and RNA modification genes

The correlation of *ORC6* expression with DNA promoter methylation levels in cancer was explored by UALCAN(https://ualcan.path.uab.edu/) (15). The correlation of *ORC6* expression with marker gene expression associated with three classes of RNA modifications (N1-methyladenosine (m1A), 5-methylcytosine (m5C) and N6-methyladenosine (m6A)) (16) across cancers was assessed using Spearman correlation analysis.

### 2.5 Identification of corresponding characteristics of *ORC6*

To clarify the expression of *ORC6* and immune-related characteristics, we employed Spearman correlation analysis to calculate the correlation between *ORC6* and 5 types of immune-related genes (chemokines, chemokine receptors, immunosimulators, immunoinhibitors, and MHC). TISIDB(http://cis.hku.hk/TISIDB/) (17) to assess the immune cell infiltration status of *ORC6*.

To assess the impact of immunotherapy on ORC6 expression, we analyzed the immunotherapy advanced urothelial carcinoma cohort (IMvigor210 cohort) (18). The R package ‘limma’ was utilized for differential expression analysis of the target gene in the different groups. Additionally, we accessed the CAMOIP database (https://www.camoip.net/) (19) to obtain the prognostic information of the Auslander-Melanoma (20) immunotherapy cohort and assessed the effect of *ORC6* expression on the survival time of patients after immunotherapy.

The possibility of *ORC6* expression as a predictive marker for immunotherapy response was analyzed using the TISMO (http://tismo.cistrome.org/) (21) and TIDE (http://tide.dfci.harvard.edu/) (22) databases. To examine the correlation between *ORC6* expression and the half-inhibitory concentration (IC50) of the drug, we employed the R package ‘pRRophetic’ (23) for the analysis.

### 2.6 Single-cell and bulk transcriptome sequencing analysis

Tumor Immune Single-cell Hub (TISCH, http://tisch.comp-genomics.org/) is a scRNA-seq database that has been specifically developed to investigate the single-cell landscape of the tumor microenvironment (TME) (24). We screened single-cell datasets, including ALL_GSE132509, BRCA_GSE161529, CESC_GSE168652, CHOL_GSE138709, CRC_GSE166555, ESCA_GSE160269, HNSC_GSE103322, LIHC_GSE166635, LSCC_GSE150321, OV_GSE154600, PAAD_ CRA001160, PRAD_GSE141445, STAD_GSE134520, THCA_GSE148673 and UVM_GSE139829. UMAP plots were used for the visualization of cell types and *ORC6* expression levels.

### 2.7 Functional enrichment analysis

We utilized single-cell sequence data obtained from CancerSEA(http://biocc.hrbmu.edu.cn/CancerSEA/) (25) to examine the relationship between *ORC6* and 14 distinct cancer functional states.

To investigate the mechanisms underlying the impact of *ORC6* expression on the prognosis of tumor patients, we performed gene set enrichment analysis (GSEA) to explore the *ORC6*-related signaling pathways, as previously described in the literature (26, 27). We performed differentially expressed gene (DEG) analysis on the *ORC6*-low and *ORC6*-high subgroups of each cancer using the “limma” R package. The threshold was set at 30%, and genes with adjusted P values <0.05 were considered DEGs. We selected the h.all.v7.2.symbols.gmt gene set as our reference and employed it to determine the normalized enrichment score (NES) and false discovery rate (FDR). By examining the correlation between the *ORC6* gene expression matrix and the known functional genome, we evaluated the impact of coordinated changes in genes within the genome on phenotypic alterations. The presented findings were visualized as bubble plots with the aid of the R package “ggplot2”. The CAMOIP (19) network server was employed to perform Kyoto Encyclopedia of Genes and Genomes (KEGG) and Gene Ontology (GO) analyses based on *ORC6* expression in TCGA-LIHC transcriptome data using the R package “clusterProfiler”.

### 2.8 Cell lines and *ORC6* expression detection

The LIHC cell lines HCCLM3 and MHCC97-H and the hepatic epithelial cell line THLE-2 were acquired from BeNa Culture Collection. The HepG2, U-251 MG, and LN229 cell lines were obtained from Procell and cultured according to the manufacturer’s instructions. Transfection was carried out in 6-well plates (NEST Biotechnology) using Lipofectamine 2000 (Invitrogen) according to the manufacturer’s protocol in HepG2, HCCLM3, U-251 MG, and LN229 cells. The siRNA used in this study was synthesized by GenePharma. **Supplementary Table S1** lists the sequences of the siRNAs used in this study. The Western blot experimental steps were described in a previous study (28). The antibodies used in this study were anti-*ORC6* (Proteintech, 17784-1-AP, 1:1000) and anti-alpha tubulin (Proteintech, 11224-1-AP, 1:5000).

### 2.9 Cell viability and proliferation assays

Control and experimental cells were placed in 96-well plates at cell densities of 5,000 (HepG2 and HCCLM3) or 3,000 (U251 MG and LN229) cells per well, respectively. After incubation for 0, 24, 48, and 78 hours, cell viability was assessed by using the CCK-8 assay (GlpBio), and the optical density (OD) was measured at 450 nm with a microplate reader.

Cell proliferation was assessed using EdU (5-ethynyl-2’-deoxyuridine) staining. Briefly, control and experimental cells were seeded in 96-well plates and incubated overnight. After incubation with 10 μM EdU (RiboBio) for 2 hours, cells were fixed with 4% paraformaldehyde for 20 minutes, permeabilized with 0.5% Triton X-100 for 15 minutes, incubated with EdU reaction solution for 30 minutes, and finally incubated with Hoechst 33342 for 10 minutes. Images were taken using an inverted fluorescence microscope (Olympus).

### 2.10 Cell migration assays

Control and experimental cells were seeded onto a 6-well plate and cultured until reaching a confluence of 70% before undergoing transfection and continuing to be cultured normally. Scratch assays were performed when cell confluence reached approximately 90%, with images taken at 0 and 36 hours thereafter. For Transwell migration assays, 50,000 (HepG2 and HCCLM3) or 15,000 (U251 MG and LN229) cells were seeded into the chamber and cultured with serum-free medium within the insert and with 10% complete medium outside of the insert. After 24 hours, cells were fixed and stained, and then the cells within the insert were removed by gently swabbing with a cotton tip before imaging.

### 2.11 Statistical analysis

We used Student’s t-test to determine the statistical significance of differences between the two groups. Paired t-tests were conducted to compare the expression levels of *ORC6* in tumor tissues with those in their paired normal tissues. We evaluated the prognostic significance of *ORC6* by conducting log-rank and univariate Cox regression analyses. Spearman correlation analysis was employed to assess the correlations between *ORC6* and its corresponding features. A p value < 0.05 was considered to indicate statistical significance.

## 3 Results

### 3.1 Expression landscape of *ORC6*

**Figure 1:**
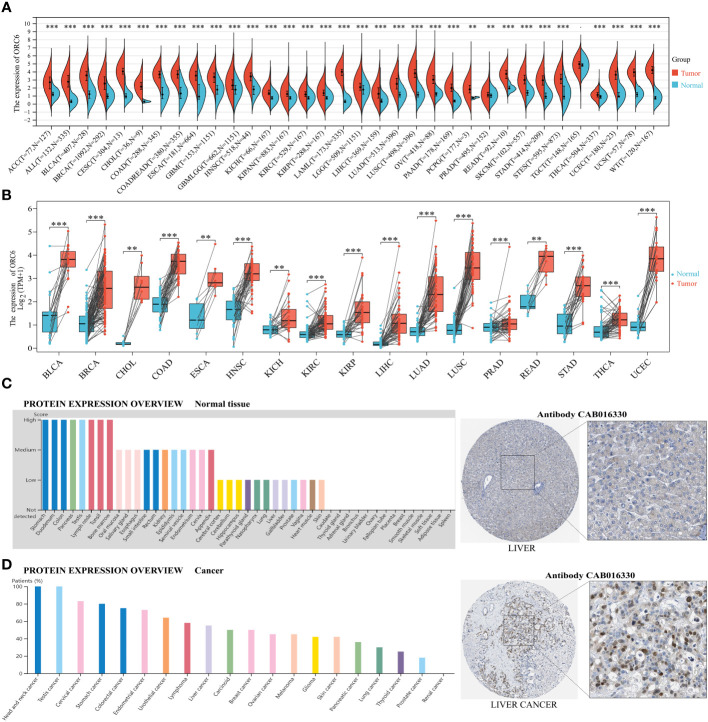
*ORC6* expression profiles. **(A)** *ORC6* mRNA expression levels in pan-cancer tissues and corresponding normal tissues derived from TCGA and GTEx databases. **(B)** Differential expression of *ORC6* in tumor and paired adjacent tissues. **(C, D)** Protein expression analysis of *ORC6* in normal tissues and cancer tissues using the Human Protein Atlas database. The left panel shows the expression scores or positive percentages in each tissue, while representative immunohistochemistry images of normal liver tissue and liver cancer tissue are shown in the right panel. (**p < 0.01, ***p < 0.001).

We conducted a comparative analysis of *ORC6* expression in tumor vs. normal tissues by merging the TCGA and GTEx databases. *ORC6* was found to be significantly upregulated in 33 tumors (all p<0.05), as illustrated in **Figure 1A**. However, no significant changes were observed in TCGT. Then, our analysis of tumor and matched normal tissue samples from the TCGA database revealed that *ORC6* expression was significantly elevated in tumor samples from BLCA, BRCA, CHOL, COAD, ESCA, HNSC, KICH, KIRC, KIRP, LIHC, LUSC, LUAD, PRAD, READ, STAD, THCA, and UCEC (**Figure 1B**, all p<0.05). Further protein score hints were provided by the HPA online database. *ORC6* showed high protein scores in the stomach, duodenum, colon, pancreas, lymph nodes, testis, and bone marrow but low protein scores in the liver (**Figure 1C**). Regarding its protein expression in tumors, we observed that *ORC6* was moderately/highly expressed in 100% of head and neck cancer (3/3) and testicular cancer (11/11) tissues and was moderately/highly expressed in 54.5% (6/11) of liver cancer tissues (**Figure 1D**). To clarify the localization of *ORC6* protein expression, we obtained immunofluorescence staining images of *ORC6* protein expression in HEK 293 and PC-3 cells through the HPA database (**Supplementary Figure S1A**). Further access to the Genecards database was performed for validation (**Supplementary Figure S1B**). We found that *ORC6* was mainly concentrated in the nucleus and cytoplasm. Finally, we constructed a PPI network using interaction data sourced from the ComPPI website. The results of our analysis showed that proteins found to be in close interaction with *ORC6* were primarily localized within the nucleus, as depicted in **Supplementary Figure S1C**. In summary, we identified that *ORC6* was generally highly expressed in tumors.

### 3.2 *ORC6* genetic alterations and epigenetic modifications

The frequency and type of *ORC6* gene genetic alterations across cancers were analyzed by the cBioPortal platform. As illustrated in **Supplementary Figure S1D**, the most frequent type of genetic alteration in *ORC6* was “amplification”, followed by “deep deletion”, “mutation” and “structural variation”. SARC exhibited the greatest frequency of *ORC6* genetic mutations. These alterations included “deep deletions” in 2.35% of genes and “structural variants” in 0.39% of genes. In PRAD, the gene alteration frequency of *ORC6* was 2.63%, of which the frequency of “amplification” reached 2.43%. In both DLBC and UCS, *ORC6* genetic alterations were “deeply deleted”. In UCEC and SKCM, the frequency of *ORC6* gene “mutation” reached 1.13%. In five different cancer types (ACC, ESCA, LIHC, KIRP, and PAAD), the *ORC6* gene only exhibited “amplified” genetic variants. The mutation frequency of the *ORC6* gene is generally low, at less than 3%. This could be due to the high conservation of genes within the ORC family (29). Subsequently, to explore the relationship between *ORC6* expression and genomic variations across different cancer types, we employed either the Wilcoxon rank-sum test or the Kruskal–Wallis rank-sum test. We observed differential expression of *ORC6* across three distinct variant groups (gain-variant, loss-variant, and no-variant) in 14 different cancers: BLCA, BRCA, CESC, COAD, HNSC, KIPAN, LIHC, LUAD, LUSC, OV, PRAD, STAD, STES, and UCS (**Supplementary Figure S1E**, all p<0.05). Specifically, *ORC6* expression was generally higher in the gain-variant group than in the loss/neutral-variant group.

Numerous reports suggest that abnormal DNA methylation in the promoter region of genes can induce changes in chromatin structure and DNA stability, ultimately leading to the dysregulation of gene expression within the body (30). Therefore, we analyzed differences in the DNA promoter methylation levels of *ORC6* between tumor and normal tissues using UALCAN. As depicted in **Supplementary Figure S2A**, methylation levels were lower in BLCA, BRCA, HNSC, THYM, UCEC, and PRAD than in normal tissues (all p<0.05). In contrast, methylation levels were higher in PAAD, KIRC, LUSC, and SARC than in normal tissues (**Supplementary Figure S2A**, all p<0.05). Moreover, RNA modifications are critical in selectively regulating the expression of genes (31). Our analysis, as illustrated in **Supplementary Figure S2B**, reveals a strong positive correlation between *ORC6* expression and m1A-, m5C-, and m6A-related genes across almost all tumor types. These findings suggest that the ubiquitous overexpression of *ORC6* in tumors may be closely associated with its epigenetic modifications and genetic variations. This correlation further supports the potential of *ORC6* as a cancer regulatory factor and provides valuable clues for further exploring its role in cancer.

### 3.3 Correlation of *ORC6* expression with clinicopathological features

**Figure 2:**
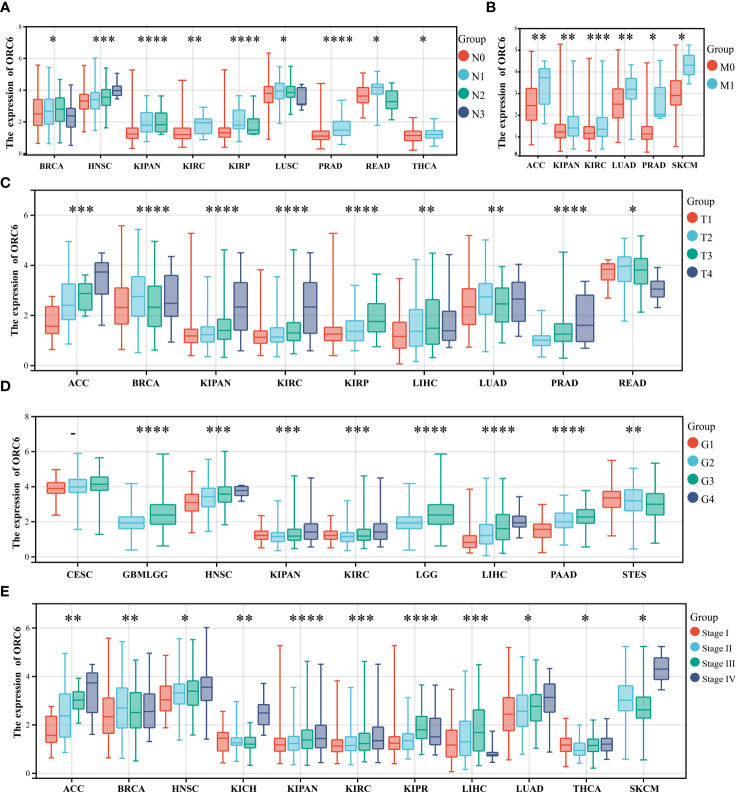
Correlations between clinical features and *ORC6*. **(A–E)** Correlation of *ORC6* expression with pan-cancer clinical N stage **(A)**, M stage **(B)**, T stage **(C)**, histological grade **(D)**, and clinical stage **(E)**. (*p<0.05, **p<0.01, ***p<0.001, ****p<0.0001).

We also investigated the correlations between *ORC6* expression and various clinicopathological features. According to the results presented in **Figure 2A**, there was a positive correlation between *ORC6* expression and lymph node metastasis in several tumor types, and the correlations in HNSC (p=2.2e-4), KIPAN (p=6.8e-8), KIRC (p=5.6e-3), PRAD (p=4.0e-8), THCA (p=0.02) and other tumor types were the most robust. **Figure 2B** shows that the increase in *ORC6* expression was positively correlated with tumor metastasis in ACC (p=8.2e-3), KIPAN (p=1.9e-3), KIRC (p=4.3e-4), LUAD (p=9.5e-3), PRAD (p=0.01), and SKCM (p=0.03). Furthermore, increased *ORC6* expression was positively correlated with the T stage of ACC (p=4.8e-8), KIPAN (p=1.1e-9), KIRC (p=1.4e-5), KIRP (p=2.4e-8) and PRAD (p =7.0e-12) (**Figure 2C**). **Figure 2D** shows that the increased expression of *ORC6* was positively correlated with the histological grade of GBMLGG (p=9.2e-25), HNSC (p=5.6e-9), LGG (p=9.2e-25), LIHC (p=3.0e-15) and PAAD (p=2.9e-9) but negatively correlated with the histological grade of STES (p=6.3e-5). Similarly, increased *ORC6* expression was positively correlated with clinical staging (**Figure 2E**), and typical tumor types were ACC (p=6.3e-5), HNSC (p=1.5e-3), KIPAN (p=3.3e-9), KIRC (p=2.4e-4), and LUAD (p=0.01). From the above findings, it can be inferred that *ORC6* might play a role in tumor progression and metastasis.

### 3.4 Prognostic and diagnostic value of *ORC6*

**Figure 3:**
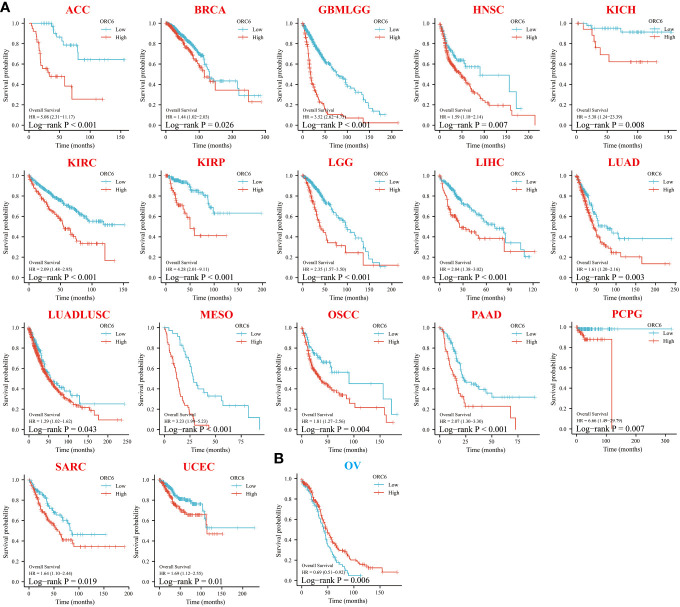
Relationship between *ORC6* expression and overall survival (OS) of patients. **(A, B)** Relationship between *ORC6* expression levels and prognosis in the indicated tumor types. In the abbreviation of tumor type, red represents poor prognosis, and blue represents good prognosis. The results are grouped by the best cutoff value, and the log-rank method was used for survival difference analysis.

To investigate the effect of *ORC6* on tumor prognosis, we plotted survival curves and assessed OS using the Kaplan–Meier method. As shown in **Figure 3**, in ACC, BRCA, GBMLGG, HNSC, KICH, KIRC, KIRP, LGG, LIHC, LUAD, LUADLUSC, MESO, OSCC, PAAD, PCPG, SARC and UCEC patients, high *ORC6* levels were highly correlated with poorer OS (**Figure 3A**; all p<0.05). Moreover, it is worth noting that high *ORC6* expression was exclusively correlated with improved OS in OV (**Figure 3B**; p<0.05).

**Figure 4:**
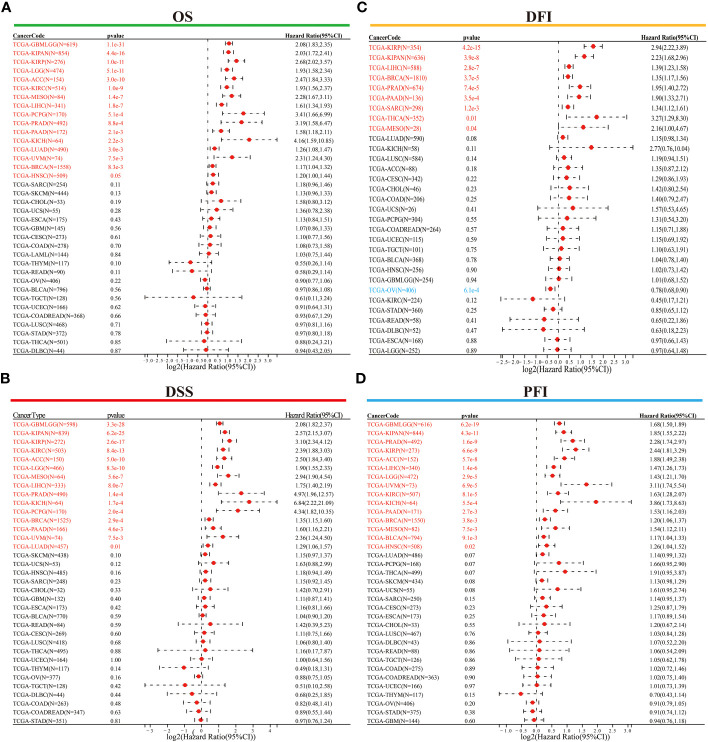
Univariate Cox regression analysis was performed to determine the prognostic role of *ORC6*. **(A–D)** Correlation of *ORC6* expression with OS **(A)**, DSS **(B)**, DFI **(C)**, and PFI **(D)**. (OS, overall survival; DSS, disease-specific survival; DFI, disease-free interval; PFI, progression-free interval).

Following this, we conducted Cox regression analysis to assess the correlation between *ORC6* expression and several survival outcomes, including OS, DSS, DFI, and PFI, for each tumor type. The results were then presented in the form of a forest plot. As shown in **Figure 4A**, our findings indicate that high *ORC6* expression was significantly associated with shorter OS in GBMLGG, KIPAN, KIRP, LGG, ACC, KIRC, MESO, LIHC, PCPG, PRAD, PAAD, KICH, LUAD, UVM, BRCA and HNSC patients (all p<0.05). High *ORC6* expression in GBMLGG, KIPAN, KIRP, KIRC, ACC, LGG, MESO, LIHC, PRAD, KICH, PCPG, BRCA, PAAD, UVM, and LUAD patients was associated with poorer DSS (**Figure 4B**, all p<0.05). Regarding DFI, there was a significant association between high *ORC6* expression and lower DFI in KIRP, KIPAN, LIHC, BRCA, PRAD, PAAD, SARC, THCA, MESO, and LUAD patients, whereas high *ORC6* expression in OV patients was associated with improved prognosis for DFI (**Figure 4C**, all p<0.05). Furthermore, as illustrated in **Figure 4D**, high *ORC6* levels were strongly correlated with poorer PFI in GBMLGG, KIPAN, PRAD, KIRP, ACC, LIHC, LGG, UVM, KIRC, KICH, PAAD, BRCA, MESO, BLCA and HNSC (all p<0.05).

**Figure 5:**
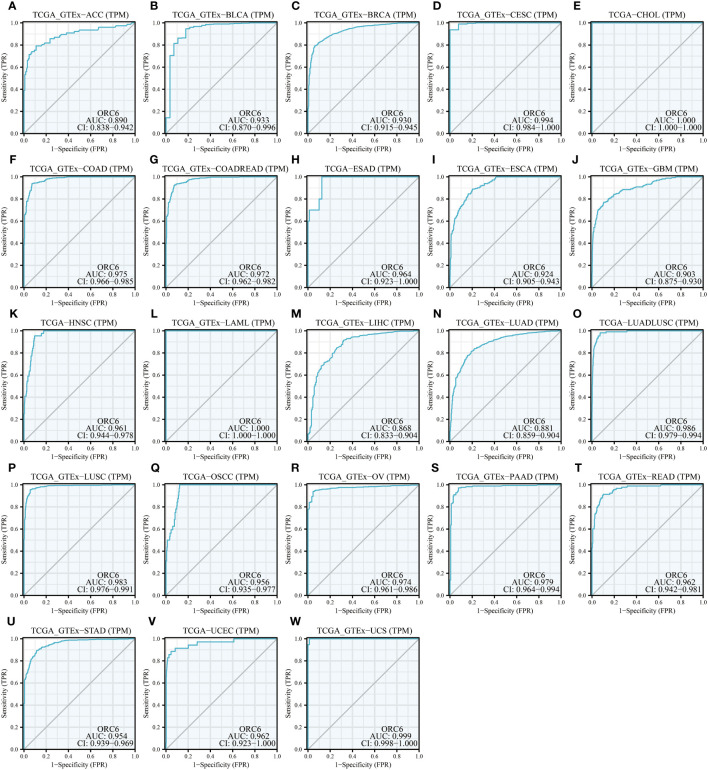
Diagnostic value of *ORC6*. **(A–W)** ROC curve of *ORC6* in ACC, BLCA, BRCA, CESC, CHOL, COAD, COADREAD, ESAD, ESCA, GBM, HNSC, LAML, LIHC, LUAD, LUADLUSC, LUSC, OSCC, OV, PAAD, READ, STAD, UCEC and UCS.

Moreover, we assessed the diagnostic accuracy of *ORC6* in different types of cancer using ROC curves. As shown in **Figure 5**, in ACC, BLCA, BRCA, CESC, CHOL, COAD, COADREAD, ESAD, ESCA, GBM, HNSC, LAML, LIHC, LUAD, LUADLUSC, LUSC, OSCC, OV, PAAD, READ, STAD, UCEC and UCS, *ORC6* could be used as a highly accurate diagnostic marker (**Figures 5A–W**, all AUC>0.85). In DLBC, GBMLGG, KICH, KIRC, KIRP, SKCM, and THYM, *ORC6* had moderate diagnostic performance (**Supplementary Figures S3A–E, H, K**; all AUC=0.7~0.85). In LGG, PRAD, TGCT, and THCA, *ORC6* had poor diagnostic values (**Supplementary Figures S3F, G, I, J**; all AUC=0.5~0.7). In conclusion, our study revealed that high expression of *ORC6* is generally linked to unfavorable prognosis in the majority of cancer types, and it has good diagnostic value.

### 3.5 Pathways and functions associated with *ORC6* expression

**Figure 6:**
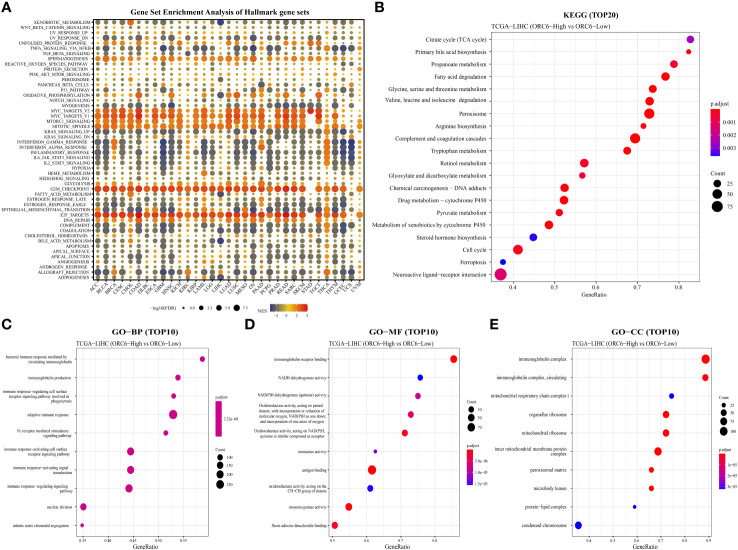
Gene set differential enrichment analysis of *ORC6* across cancers and in liver cancer. **(A)** This graph visualizes the potential signaling pathways that are associated with *ORC6* expression in different tumor types. The circles depict cancer enrichment projects. Their sizes vary based on their false discovery rate (FDR), while the colors represent the corresponding normalized enrichment score (NES) for each enrichment item. **(B)** The top 20 enriched KEGG results in HCC are shown. **(C–E)** The top 10 enriched gene ontology biological processes (BPs) **(C)**, molecular functions (MFs) **(D)**, and cellular components (CCs) **(E)** are also displayed.

To explore the possible biological pathways influenced by *ORC6* that may contribute to tumorigenesis and progression, we conducted GSEA on data obtained from 33 tumors from TCGA. As illustrated in **Figure 6A**, we observed that immune-related pathways, including TNFα signaling via NFκB, IFN-α response, IFN-γ response, inflammatory response, IL-6/JAK/STAT3, IL-2/STAT5, complement and coagulation cascades, and allograft rejection pathways, were significantly enriched across a diverse range of tumors. Moreover, we observed a positive correlation between *ORC6* expression and MYC target V2, MYC target V1, MTORC1, mitotic spindle, G2 checkpoint, E2F target, DNA repair, and other pathways across cancers. Furthermore, KEGG analysis revealed that *ORC6* was mainly involved in the synthesis and degradation of various substances, drug metabolism, the cell cycle, ferroptosis, and neuroactive ligand-receptor interactions in LIHC (**Figure 6B**). GO analysis, including the BP, CC, and MF categories, indicated that *ORC6* was mainly related to immune response regulation and biological enzyme activity in LIHC (**Figures 6C–E**).

**Figure 7:**
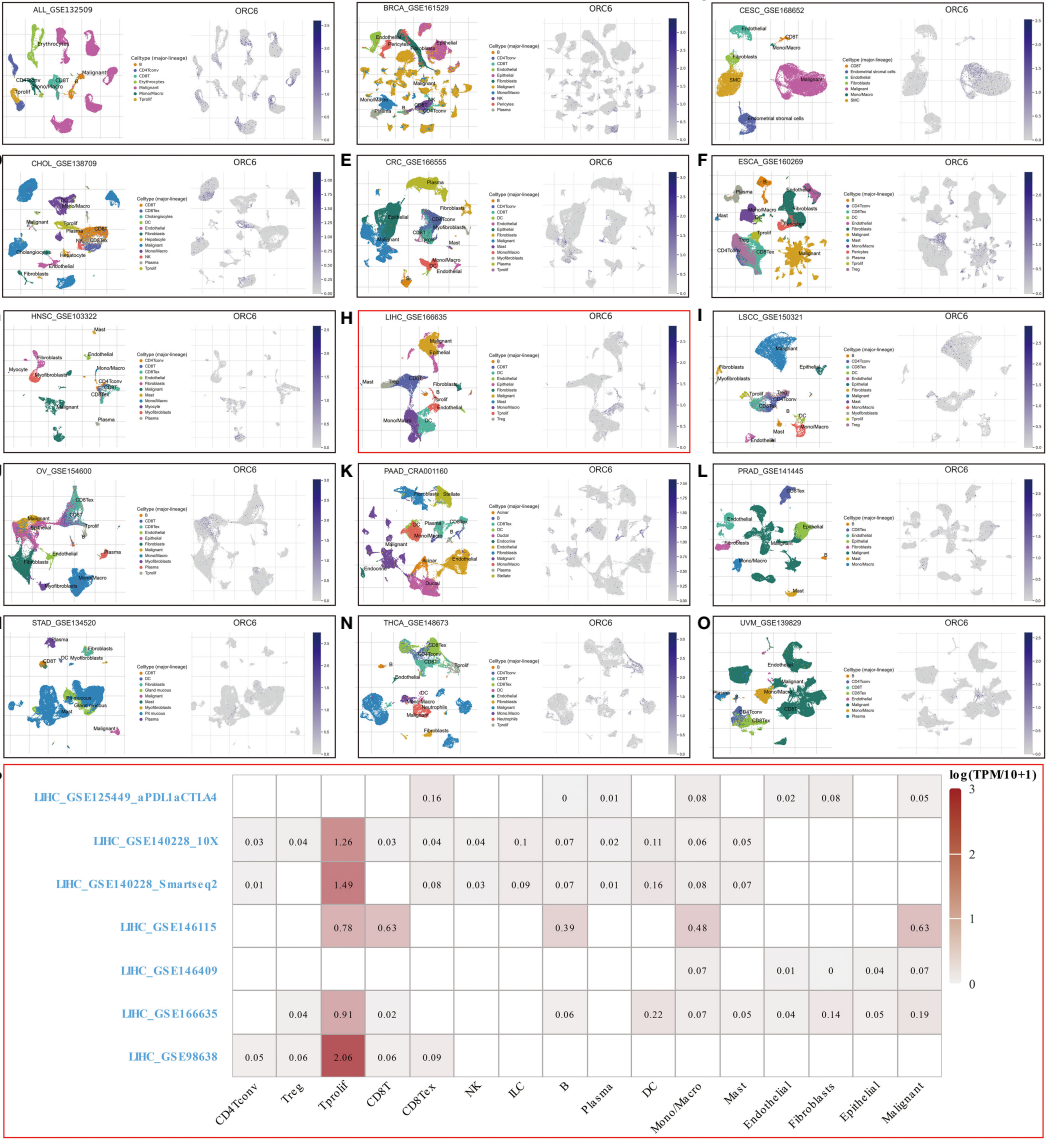
Single-cell sequencing analysis of *ORC6* expression in malignant cells. **(A–0)** *ORC6* in ALL **(A)**, BRCA **(B)**, CESC **(C)**, CHOL **(D)**, CRC **(E)**, ESCA **(F)**, HNSC **(G)**, LIHC **(H)**, LSCC **(I)**, OV **(J)**, PAAD **(K)**, PRAD **(L)**, THCA **(N)** and UVM **(O)** were mainly expressed in malignant cells; *ORC6* was mainly expressed in pit mucous in STAD **(M)**. **(P)** Expression distribution of *ORC6* in several LIHCs.

To explore *ORC6* expression in diverse TMEs, including ALL, BRCA, CESC, CHOL, CRC, ESCA, HNSC, LIHC, LSCC, OV, PAAD, PRAD, STAD, THCA, and UVM, we investigated their expression distribution (**Figures 7A–O**). The results were interesting, as they showed that *ORC6* was primarily expressed at high levels in the malignant cells of these cancers. In STAD, it was predominantly expressed in pit mucus cells (**Figure 7M**). It is worth noting that in LIHC, *ORC6* was also found to be expressed in T-cell proliferation, which demonstrates its potential role in this immune response (**Figures 7H, P**). To further investigate the relationship between *ORC6* and the functional status of different cancers, we analyzed single-cell sequencing data obtained from CancerSEA for 14 types of cancer. In most tumors, *ORC6* showed a positive correlation with the cell cycle, proliferation, DNA damage, and DNA repair (**Supplementary Figure S4**). In contrast, *ORC6* was negatively associated with apoptosis, hypoxia, metastasis, and quiescence in most tumors (**Supplementary Figure S4**). These findings suggest a correlation between abnormal expression of *ORC6* and the advancement of cancer as well as the immune response of cancer.

### 3.6 Correlation between *ORC6* expression and the tumor immune landscape

**Figure 8:**
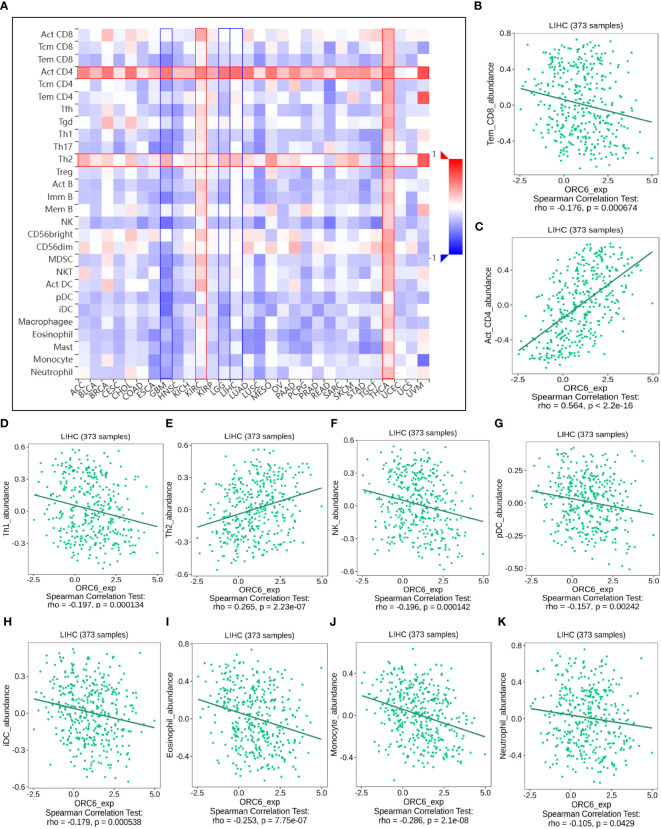
The relationship between *ORC6* levels and immune infiltration was analyzed by Timer 2.0. **(A)** *ORC6* expression correlates with immune cell infiltration across cancers. **(B–K)** Correlation of *ORC6* expression with the abundance of Tem CD8, Act CD4, Th1, Th2, NK, pDC, iDC, eosinophil, monocyte and neutrophil cell infiltration in LIHC.

Investigating the possible gene expression within tumors and its connection to immune cells can greatly aid in predicting the clinical outcome for patients with tumors and selecting appropriate diagnostic targets and intervention strategies (32). To gain further insights into the correlation between *ORC6* and immune cells in the TME, we used the TISIDB tool for analysis. The pan-cancer analysis indicated that the expression level of *ORC6* displayed an inverse correlation with the infiltration abundance of various immune cells, including Tem CD8 cells, Th1 cells, NK cells, pDCs, iDCs, eosinophils, monocytes, and neutrophils, while showing a positive correlation with the infiltration abundance of Act CD8 cells and Th2 cells (**Figure 8A**). This phenomenon was especially evident in LIHC (**Figures 8B–K**). Furthermore, in gliomas, which include GBM and LGG, *ORC6* expression was negatively correlated with the abundance of infiltrating immune cells, including Tem CD8, Tcm CD4, Tfh, Th1, Th17, Act B, lmm B, NK, MDSC, NKT, Act DC, pDC, iDC, macrophage, eosinophil, mast, monocyte, and neutrophil cells. In contrast, *ORC6* expression levels in THCA and KIRC were positively correlated with the majority of immune cell infiltration (**Figure 8A**).

**Figure 9:**
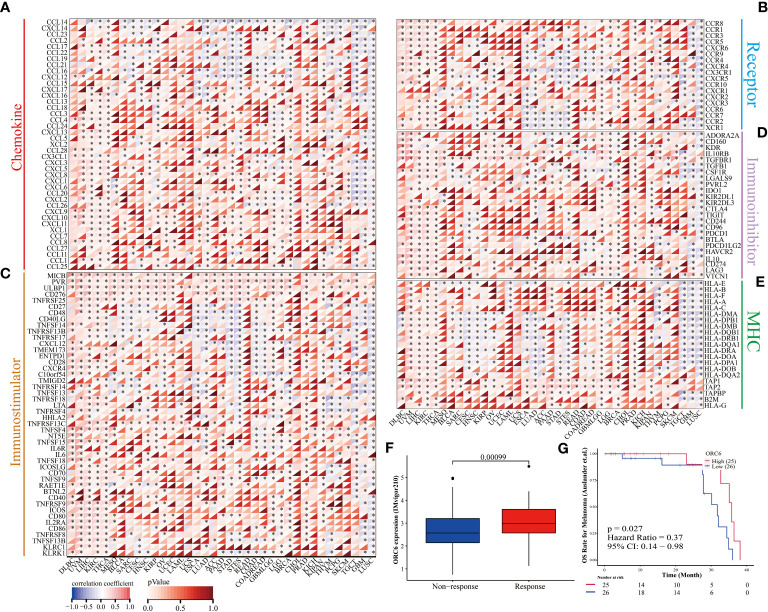
*ORC6* expression and immunotherapy response. **(A–E)** Correlation analysis of *ORC6* expression with five types of immune regulation-related genes, including chemokine **(A)**, chemokine receptor **(B)**, immune stimulator **(C)**, immune inhibitors **(D)**, and MHC **(E)**. **(F)** Association of *ORC6* expression with immunotherapy response in the IMvigor210 cohort. **(G)** In the melanoma immunotherapy cohort, the log-rank method was used to analyze the difference in survival after immunotherapy between the high- and low-expression groups of *ORC6*. *p<0.05.

Furthermore, we examined the association between *ORC6* expression and the expression of genes related to immune regulation (**Figures 9A–E**). The heatmap indicated that *ORC6* was coexpressed with most immune-related genes across cancers. Especially in DLBC, UVM, LIHC, KIRC, and THCA, *ORC6* was roughly positively correlated with 5 immune-related genes. In TGCT, GBM, and LUSC, *ORC6* was roughly negatively correlated with immune-related genes. Furthermore, the chemokine CCL14 was negatively correlated with *ORC6* expression across cancers (**Figure 9A**). Among the immune activation-related genes, MICB, PVR, ULBP1, CD276, and TNFRSF25 were positively correlated with *ORC6* expression in most tumors (**Figure 9C**). There was a positive correlation between the expression of *ORC6* and genes related to immunosuppression in several cancer types, such as DLBC, UVM, LIHC, KIRC, THCA, GBMLGG, LGG, and PRAD, as indicated in **Figure 9D**. Notably, in the advanced urothelial carcinoma cohort with immunotherapy, the response group had significantly higher *ORC6* expression (**Figure 9F**, p=0.00099). Furthermore, we observed that in the melanoma immunotherapy cohort, the high *ORC6* expression group had prolonged survival after immunotherapy (**Figure 9G**, p=0.027). Taken together, these results suggest that *ORC6* may be involved in immune cell infiltration and the expression of immunomodulatory genes and that high *ORC6* expression may indicate a better response to immunotherapy.

### 3.7 *ORC6* predicts immunotherapy response and chemotherapy efficacy

**Figure 10:**
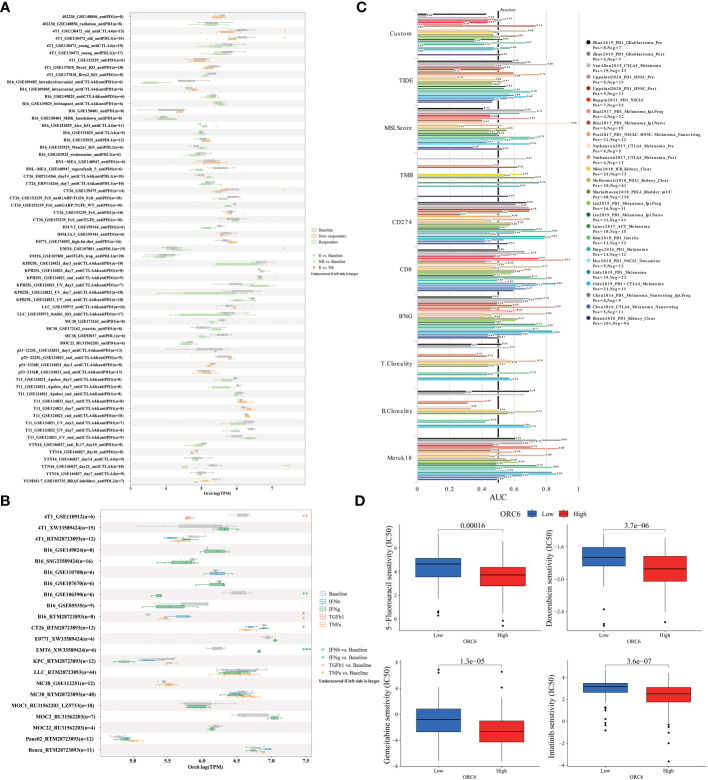
Correlation of *ORC6* expression with immunotherapy response, biomarkers, and drug sensitivity. **(A)** Differences in *ORC6* expression between the immunosuppressive treatment group and the control group. **(B)** Differences in *ORC6* expression between cytokine-treated and control groups. **(C)** The predictive role of *ORC6* as a biomarker versus other markers in different immunotherapy cohorts. **(D)** Relationship between *ORC6* expression and half-inhibitory concentration (IC50) of 5-fluorouracil, doxorubicin, gemcitabine and imatinib. *p < 0.05, **p < 0.01, ***p < 0.001.

To elucidate the predictive function of *ORC6* expression in immunotherapy response, we evaluated it using the TISMO database. As depicted in **Figure 10A**, *ORC6* expression was markedly different in 5 subjects before and after ICB treatment and between responder and nonresponder cohorts. Moreover, *ORC6* expression was significantly different in the six cell lines before and after cytokine treatment (**Figure 10B**). Furthermore, we performed a biomarker assessment of *ORC6* by TIDE. The findings indicated that *ORC6* had a better predictive effect in 7 immunotherapy cohorts (**Figure 10C**). In addition, we performed a sensitivity analysis of chemotherapy drugs commonly used to treat LIHC. As shown in **Figure 10D**, the *ORC6* high-expression group was closely correlated with the reduction of IC50 of 5-fluorouracil, doxorubicin, gemcitabine, and imatinib (all p<0.001).

### 3.8 External cohort and *in vitro* experiments clarify the promotional effect of *ORC6* on LIHC and GBMLGG

After collating and analyzing the pan-cancer data mentioned above, it was observed that the elevated expression of *ORC6* was significantly associated with the unfavorable prognosis and malignancy of LIHC and GBMLGG patients (**Figures 1**–**4**). Consequently, our study will concentrate on LIHC and GBMLGG.

**Figure 11:**
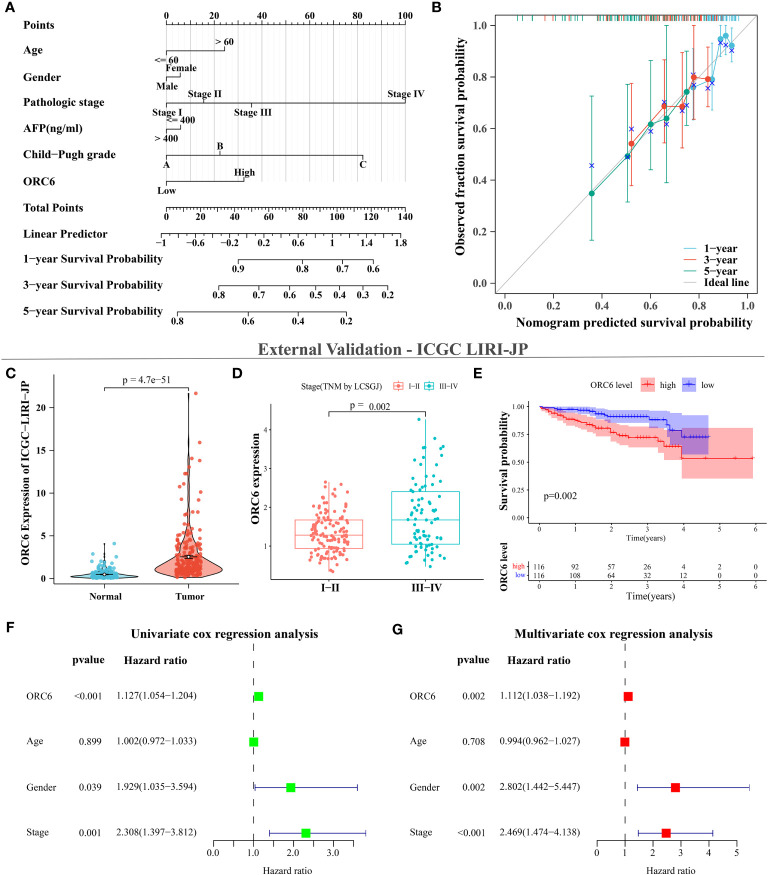
*ORC6* is an independent risk factor for poor prognosis in LIHC. **(A)** Construction of line charts for 1-, 3-, and 5-year time points. **(B)** Calibration curves for 1-, 3-, and 5-year timepoints. **(C–G)** External data validation using the ICGC LIRI-JP cohort. **(C)** Differential expression analysis of *ORC6* between tumor and normal tissues. **(D)** Differential expression analysis of *ORC6* across different clinical stages. **(E)** Survival analysis of the high and low *ORC6* expression groups, with both univariate **(F)** and multivariate **(G)** Cox regression analyses used to establish the role of *ORC6* in LIHC.

**Figure 12:**
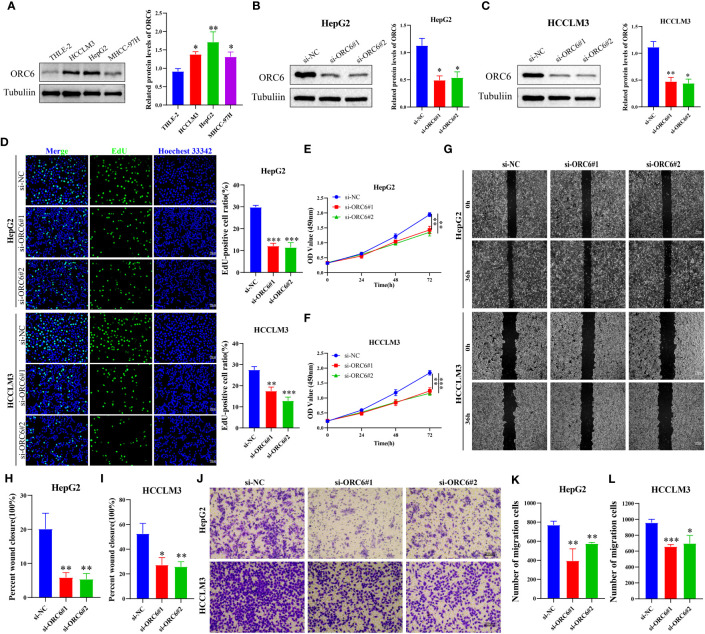
Silencing *ORC6* expression suppresses the proliferation and migration of LIHC cells. **(A)** Western blot analysis and quantitative measurements of *ORC6* protein levels in liver cells (THLE-2) and LIHC cells (HCCLM3, HepG2, and MHCC-97H). Western blot analysis and quantitative measurements of *ORC6* knockdown efficiency in HepG2 **(B)** and HCCLM3 **(C)** cells. **(D)** EdU staining and quantitative analysis were performed to evaluate changes in cell proliferation following *ORC6* knockdown. A CCK-8 assay was utilized to evaluate the effect of *ORC6* knockdown on cell viability in HepG2 **(E)** and HCCLM3 **(F)** cells. **(G–I)** A wound-healing assay was used to evaluate the changes in the cell migration rate among the si-NC, si-*ORC6*#1, and si-*ORC6*#2 groups of HepG2 and HCCLM3 cells. **(J–L)** Transwell assays were utilized to evaluate the changes in cell migration ability among the si-NC, si-*ORC6*#1, and si-*ORC6*#2 groups in HepG2 and HCCLM3 cells. *p < 0.05, **p < 0.01, ***p < 0.001.

We collected clinical information and *ORC6* expression profiles of patients belonging to the TCGA-LIHC cohort. After performing a chi-square test analysis, we discovered a significant correlation between *ORC6* expression and tumor histological grade, alpha-fetoprotein (AFP) content, and vascular invasion in LIHC (**Supplementary Table S2**). Furthermore, via univariate and multivariate Cox regression analysis, we identified *ORC6* and pathological stage as independent prognostic risk factors for LIHC (**Supplementary Table S3**). Subsequently, a nomogram was developed to estimate the survival likelihood of patients at intervals of 1, 3, and 5 years, and its prediction efficiency was confirmed by the calibration curve, as illustrated in **Figures 11A**, **B**. This indicates that the model had a high accuracy in its predictive ability. Given that our analysis of *ORC6* was solely based on the TCGA database, we conducted external verification by collating clinical information and *ORC6* expression profiles of patients belonging to the ICGC-LIRI-JP cohort. The results showed that *ORC6* expression in LIHC was significantly higher than that in normal tissues (**Figure 11C**). It was positively correlated with the clinical stage and associated with poor overall survival (**Figures 11D, E**). Furthermore, univariate and multivariate Cox regression analyses revealed *ORC6*, sex, and clinical stage as independent risk factors for poor prognosis in LIHC (**Figures 11F, G**). To investigate the function of *ORC6* in LIHC cells, we carried out *in vitro* experiments. Initially, we assessed the basal expression of *ORC6* in LIHC cell lines and normal hepatocytes. Our findings indicate that LIHC cell lines have noticeably increased *ORC6* expression compared to normal hepatocytes, as illustrated in **Figure 12A**. To assess the impact of *ORC6* downregulation in LIHC cells, we chose two LIHC cell lines with high expression (HepG2 and HCCLM3) and conducted an *ORC6* knockout assay (**Figures 12B, C**). Using the EdU cell proliferation test, a significant decrease in the proliferation of LIHC cells was observed after the knockout of *ORC6* expression (**Figure 12D**). In addition, similar results were obtained through CCK-8 analysis (**Figures 12E, F**). Subsequently, we conducted wound healing and Transwell tests to examine the impact of *ORC6* downregulation on the migratory capacity of LIHC cells. The results indicate that compared to the control cells, the downregulation of *ORC6* significantly inhibited the migration ability of LIHC cells (**Figures 12G–L**).

**Figure 13:**
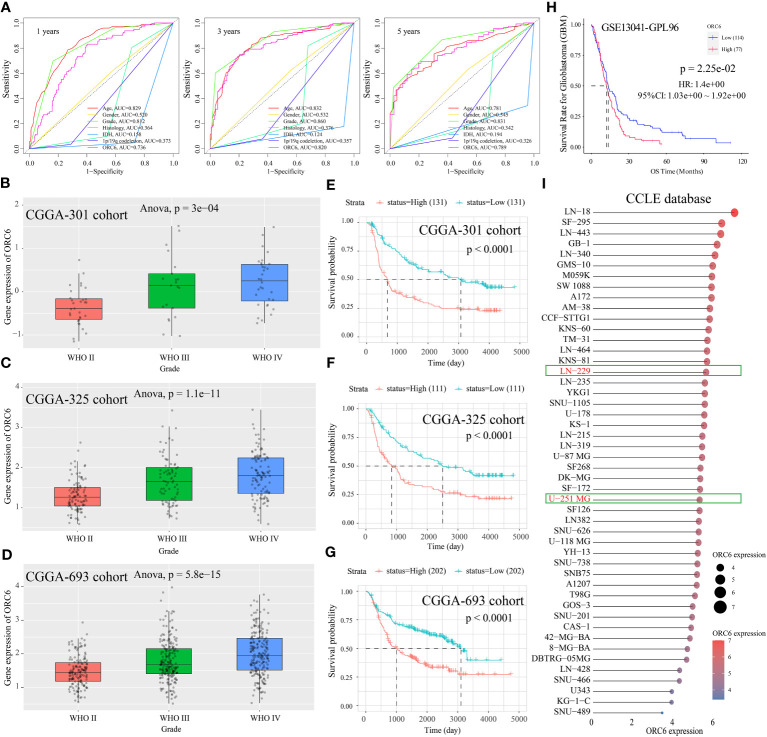
*ORC6* effectively predicts the prognosis of the patients with GBMLGG. **(A)** Receiver operating characteristic (ROC) curves for predicting 1-, 3-, and 5-year overall survival (OS) of patients with GBMLGG. **(B–D)** Analysis of differential expression of *ORC6* in different WHO grades explored in the CGGA-301, CGGA-325, and CGGA-693 cohorts, respectively. **(E–H)** Survival differences between high and low *ORC6* expression groups were examined in the CGGA-301, CGGA-325, CGGA-693 and GSE13041 cohorts. **(I)** The Cancer Cell Line Encyclopedia (CCLE) database was used to analyze the expression of *ORC6* in GBMLGG cell lines.

**Figure 14:**
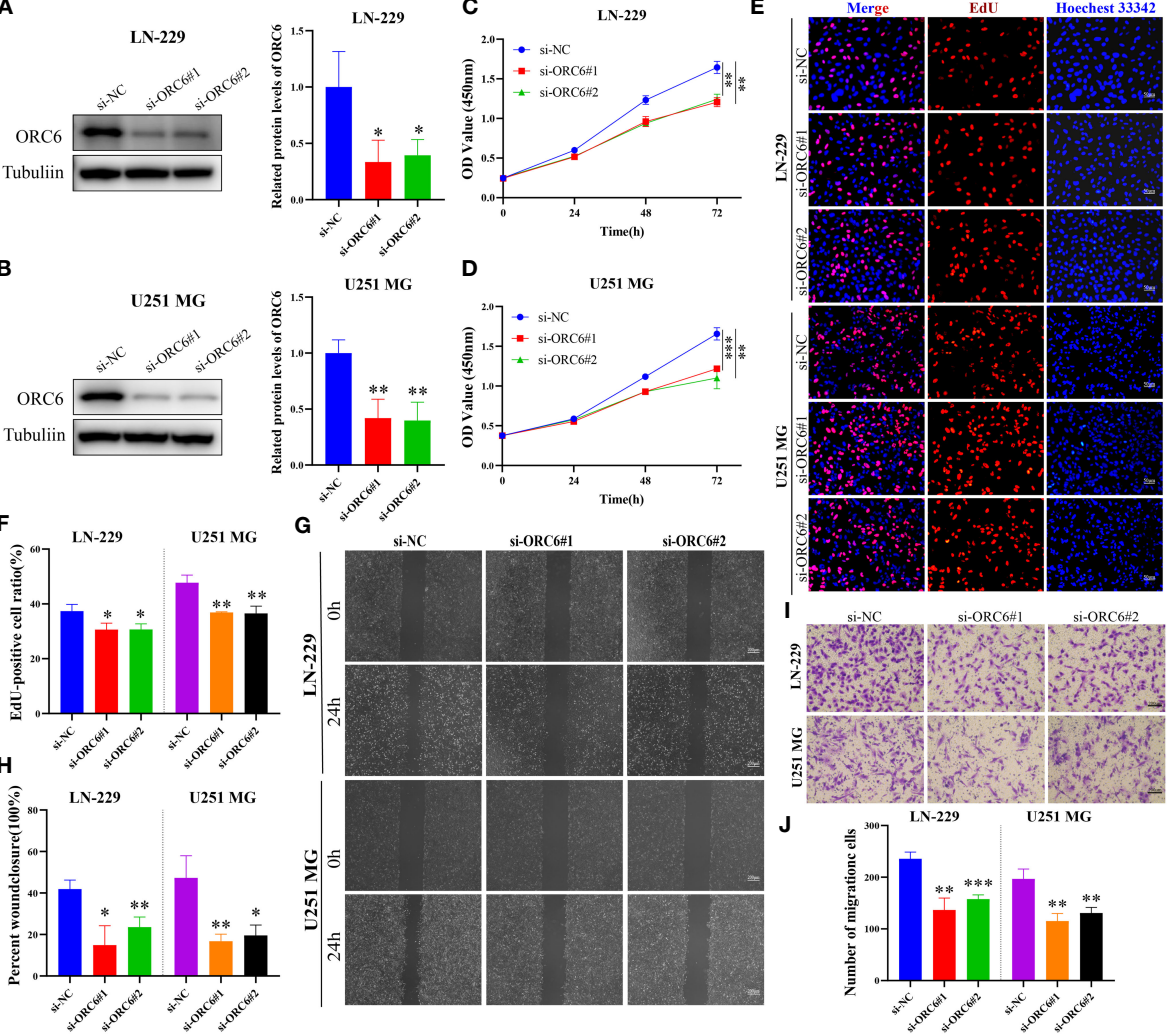
Knockdown of *ORC6* expression suppresses proliferation and migration of GBMLGG cells. **(A, B)** Western blot analysis and quantitative measurements of *ORC6* knockdown efficiency in LN-229 and U251 MG cells. **(C, D)** CCK-8 assay was used to evaluate the effect of *ORC6* knockdown on cell viability in LN-229 and U251 MG cells. **(E, F)** EdU staining and quantitative analysis were performed to evaluate the changes in cell proliferation after *ORC6* knockdown. **(G, H)** A wound healing assay was used to evaluate the changes in cell migration rate among the si-NC, si-*ORC6*#1, and si-*ORC6*#2 groups of LN-229 and U251 MG cells. **(I, J)** Transwell assays were used to evaluate the changes in cell migration ability among the si-NC, si-*ORC6*#1, and si-*ORC6*#2 groups in LN-229 and U251 MG cells. *p < 0.05, **p < 0.01, ***p < 0.001.

Subsequently, we compiled the *ORC6* expression and clinical information of patients in the TCGA-GBMLGG cohort. The chi-square test revealed a significant association between *ORC6* expression and age, histological type, WHO grade, IDH status, and 1p/19q codeletion in GBMLGG patients (**Supplementary Table S4**). Furthermore, our investigation revealed that *ORC6*, age, WHO grade, and 1p/19q codeletion were independent prognostic risk factors for GBMLGG patients, as confirmed by univariate and multivariate Cox regression analyses (**Supplementary Table S5**). Time-dependent ROC curve analysis further identified age, WHO grade, and *ORC6* as the top three effective predictors for 1-, 3-, and 5-year patient survival (**Figure 13A**, all AUC>0.7). Our results were validated in an external cohort. As demonstrated in **Figures 13B–D**, *ORC6* expression increased concomitantly with WHO grade in the CGGA-301, CGGA-325, and CGGA-693 cohorts. Moreover, high levels of *ORC6* expression were significantly associated with an unfavorable prognosis among GBMLGG patients in the CGGA-301, CGGA-325, CGGA-693, and GSE13041 cohorts (**Figures 13E–H**, all *p* < 0.05). In addition, the expression profile of *ORC6* in the GBMLGG cell line was analyzed using the CCLE database (**Figure 13I**). Further, the expression of *ORC6* protein was effectively inhibited in U251 MG and LN229 cells (**Figures 14A, B**). Consistent with findings in LIHC, the inhibition of *ORC6* expression led to decreased proliferation and migration of U251 MG and LN229 cells (**Figures 14C–J**). In conclusion, our *in vitro* results strengthen the evidence supporting the carcinogenic effects of *ORC6* in LIHC and GBMLGG.

## 4 Discussion

Multi-omics data mining analysis is crucial for exploring tumor heterogeneity and complexity and identifying prognostic biomarkers. Prior studies have linked high *ORC6* expression to poor tumor prognosis, progression, and drug resistance in some cancers (33, 34), but its prognostic and biological significance in most cancer types remains unclear. We performed a comprehensive pan-cancer study of *ORC6* and revealed its important role in LIHC and GBMLGG. In addition, we verified that *ORC6* was highly expressed in LIHC and GBMLGG and could serve as an independent marker of poor prognosis. Further external cohort analysis and *in vitro* experiments supported our findings.

In this study, we observed that the expression of *ORC6* was higher in most tumors than in normal tissues, as well as in paired cancer and paracancerous tissues. Next, we analyzed the correlations between *ORC6* expression and clinicopathological features and discovered that *ORC6* expression was positively correlated with tumor size, metastasis, histological grade, lymph node metastasis, and clinical analysis, which further implied that *ORC6* expression was associated with tumor progression and metastasis. By utilizing log-rank and Cox regression analysis, it was determined that increased expression of *ORC6* was significantly linked to unfavorable prognosis in various types of tumors, in concurrence with previous research (10, 34, 35). Through ROC curves, we also found that *ORC6* was a highly accurate diagnostic marker for most tumor types. Single-cell functional analysis also indicated that *ORC6* expression was positively correlated with the cell cycle and proliferation of tumor cells. We found that *ORC6* and pathologic stage were independent prognostic risk factors for patients with LIHC. *ORC6*, age, WHO grade, and 1p/19q codeletion are independent risk factors for poor prognosis of GBMLGG, and this result is consistent with previous studies (36). Furthermore, the time-dependent ROC curves showed that *ORC6* was more accurate than sex, histological type, IDH, and 1p/19q colocation in predicting the 1-, 3-, and 5-year survival of GBMLGG patients. After silencing *ORC6* expression, we found that the proliferation and migration abilities of LIHC and GBMLGG cells were attenuated. The results of this study suggest that elevated *ORC6* levels may serve as a valuable prognostic marker for adverse outcomes in most tumor types. However, the validation of this study was limited to *in vitro* experiments, and further *in vivo* studies are needed to fully explore this possibility.

Genetic alterations and altered epigenetic regulation are considered major factors in cancer development and progression (37, 38). In recent years, there has been growing recognition that RNA not only serves as an intermediary or effector molecule in protein synthesis but also plays a crucial and direct functional role in regulating gene expression. Consequently, the significance of RNA modifications has gained increasing prominence in scientific research and healthcare settings. Extensive evidence has suggested that the perturbation of RNA epigenetic pathways is associated with the development and progression of various human diseases, including cancer (39). In our study, we found that genetic alterations, DNA promoter methylation, and RNA modifications of *ORC6* have important effects on its expression. The main mutation forms of *ORC6* in tumors were “amplification” and “deep deletion”, and the amplification was mainly concentrated in PAAD, BRCA, OV, BLCA, ACC, ESCA, LIHC, LUAD, etc. We also noticed that *ORC6* expression was markedly linked to CNV, mainly in BRCA, CESC, HNSC, LUAD, LUSC, OV, and STES. In BLCA, BRCA, HNSC, THYM, UCEC, and PRAD, as the levels of *ORC6* promoter methylation were reduced compared to those in normal tissues. However, in PAAD, KIRC, LUSC, and SARC, there was a significant increase in *ORC6* promoter methylation levels. Furthermore, we identified a positive correlation between *ORC6* expression and m1A-, m5C-, and m6A-related genes in almost all of the analyzed tumor types. Our single-cell functional analysis also indicated a close association between *ORC6* expression and DNA damage and repair mechanisms, which highlights the underlying mechanisms of aberrant *ORC6* expression in cancer from both genetic alteration and epigenetic modification perspectives.

Cancer progression, metastasis, invasion, and resistance to therapy are modulated by bidirectional interactions between cancer cells and the TME (40). Characteristics of TME include hypoxia, immunosuppression, chronic inflammation, acidosis, high interstitial fluid pressure, increased ECM stiffness, and depletion of essential nutrients. Immunotherapy mainly targets hypoxia and immunosuppression, which are presently active research topics (41). Precision medicine aims to develop targeted and immunotherapies to enhance the survival rate. Cancer immunotherapy presents an effective and groundbreaking method to fight cancer by manipulating or modulating the immune system to elicit a robust response against tumors (42). Successful cancer immunotherapy depends on overcoming the immunosuppressive environment in the TME of cancer patients (43). Increasing evidence suggests that immune dysregulation plays a critical role in allowing tumors to evade the host immune system (44), involving both innate and adaptive immunity. Research has revealed that tumor-infiltrating lymphocytes tend to exhibit dysfunctional behavior and may remain in a quiescent state near cancerous cells. Despite this, a few patients’ T cells have been found to preserve their ability to proliferate and persist, leading to the complete eradication of sizable tumor deposits (45). This finding is consistent with our finding in single-cell sequencing that *ORC6* is predominantly expressed on Tprolif and malignant cells. Therefore, targeting ORC6 could potentially offer a precise method for identifying Tprolif and malignant cells, leading to novel avenues for tumor immunotherapy (46). Here, we found an inverse correlation between the expression level of *ORC6* and the abundance of immune cells widely believed to contribute to the suppression of tumor infiltration, including Tem CD8 cells, Th1 cells, NK cells, pDCs, iDCs, eosinophils, and monocytes (47–52). Interestingly, we noticed that *ORC6* expression levels were positively correlated with the abundance of Act CD4 and Th2 cells in the TME. Act CD4 refers to activated CD4 T cells, a key component of the adaptive immune system. Recent studies have found that CD4+ T-cell infiltration defines an immune escape environment and predicts poor patient outcomes (53). Th2 refers to helper T-cell type 2, which is a specific type of T-cell in the immune system. Th2 cells play a critical role in the adaptive immune response by supporting B-cell function. Interestingly, the accumulation of Th2 cells within tumors, in addition to Hodgkin’s lymphoma, has been associated with a poor prognosis in several types of cancers (54). At present, researchers are exploring ways to regulate Th2 cells to improve the effect of tumor treatment. Regulatory T cells (Tregs) are a subset of T cells crucial for maintaining immune homeostasis and tolerance. Research suggests that several subtypes of Tregs, including TNFR2+, LAG3+, TIM3+, and CTLA-4+ Tregs, demonstrate potent anticancer capabilities. However, in recent years, researchers have also discovered links between particular highly infiltrated Treg subtypes within tumors and favorable patient outcomes, such as CD30+OX40+ and BLIMP‐1+FOXP3+ Tregs (55). This might elucidate the favorable correlation between *ORC6* and Treg infiltration abundance in BRCA, KIRC, MESO, and THCA, whereas an inverse correlation exists in the majority of other tumor types.

Cancer cells can secrete important cytokines and chemokines for the TME during growth and progression (56), these cytokines and chemokines can in turn regulate the TME and cell signaling pathways to affect cancer progression (57, 58). Our study revealed a positive correlation between *ORC6* expression and cytokines as well as receptors in different types of tumors. Notably, GSEA revealed a strong association between *ORC6* expression and the cytokine-cytokine receptor interaction pathway, and our GSEA further highlights the close association between *ORC6* and immune-related pathways in multiple tumor types. Moreover, we discovered a positive association between *ORC6* expression and well-known targets for classical immune suppression and activation, such as PVR, MICB, ULBP1, CD276, CTLA4, TNFRSF25, PD-1 (PDCD1), TIGIT, PD-L2 (PDCD1LG2), HAVCR2, PD-L1 (CD274), and LAG3. Strikingly, our findings reveal that *ORC6* expression has a certain predictive effect on immunotherapy, cytokine therapy, and chemotherapy response. The current use of multifunctional carriers to deliver therapeutic drugs to lesion sites helps to significantly improve the effect of noninvasive treatment. Multifunctional carriers allow for multiple treatment options, including photodynamic therapy, photothermal therapy, chemotherapy, immunotherapy, or their synergistic treatments (59). Therefore, molecular probes targeting *ORC6* combined with multifunctional carriers are promising cancer treatment strategies (60). Collectively, these observations provide new insights into the complex regulation of immune cell-mediated tumor suppression and suggest that *ORC6* may serve as a promising predictive marker of immunotherapy efficacy in cancer treatment. Nevertheless, the mechanisms by which *ORC6* regulates the tumor immune microenvironment and tumor progression need to be further elucidated in the future.

## 5 Conclusion

*ORC6* emerges as a promising prognostic biomarker across various cancer types, particularly in LIHC and GBMLGG. This study underscores the correlation between high *ORC6* expression and the tumor immunosuppressive environment. These findings suggest a potential role for *ORC6* in tumor immune regulation, thereby offering further support for advancing the development of cancer immunotherapies.

## Data availability statement

The datasets presented in this study can be found in online repositories. The names of the repository/repositories and accession number(s) can be found in the article/**Supplementary Material**.

## Ethics statement

Ethical approval was not required for the study involving humans in accordance with the local legislation and institutional requirements. Written informed consent to participate in this study was not required from the participants or the participants’ legal guardians/next of kin in accordance with the national legislation and the institutional requirements.

## Author contributions

JZ conducted the formal analysis and wrote the original draft. WY performed the project administration. QC, LZ, HG, TW, YHe, and YD conducted the experiments. JX, JPang, JPeng, HG, TW, and YHan participated in software analysis. JZ, QC, LZ, and JPeng conducted data curation. JZ and WY contributed to writing, reviewing, and editing the article. WY provided funding acquisition. All authors read and approved the final submitted manuscript.

## Supplementary material

Supplementary Figure S1
*ORC6* genetic alterations, localization, and interactions. **(A)** Immunofluorescence images and merged images of ORC6 protein, nucleus, endoplasmic reticulum (ER), and microtubules in HEK 293 and PC-3 cell lines. **(B)** ORC6 expression mapping was obtained through the Genecard website. **(C)** Protein-protein interaction (PPI) network presenting proteins that interact with ORC6. **(D)** Mutation types and frequencies of ORC6 in pan-cancer were obtained from the cBioPortal website. **(E)** The expression levels of ORC6 in various CNV status in pan-cancer. *p < 0.05, **p < 0.01, ***p < 0.001.

Supplementary Figure S2
*ORC6* DNA methylation levels and the relationship between *ORC6* and RNA-modifying gene expression. **(A, B)** Promoter methylation levels of *ORC6* in different cancer types compared to normal adjacent tissues. Beta values represent DNA methylation levels ranging from 0 (unmethylated) to 1 (fully methylated). **(C)** The relationship between *ORC6* expression and gene expression related to three types of RNA modifications was analyzed by Spearman correlation. *p<0.05.

Supplementary Figure S3 Diagnostic value of *ORC6* across cancers. **(A-W)** ROC curve of *ORC6* in DLBC, GBMLGG, KICH, KIRC, KIRP, LGG, PRAD, SKCM, TGCT, THCA, and THYM.

Supplementary Figure S4 Correlation between *ORC6* expression and the functional status of 14 cancers. **(A)** The correlation between *ORC6* expression and the functional status of 14 cancers was analyzed using single-cell sequence data from the CancerSEA database.
