# Supplementary figures and images for: Multi-omics analysis reveals the involvement of origin recognition complex subunit 6 in tumor immune regulation and malignant progression
Source: Front Immunol. 2023 Oct 12;14:1236806. doi: 10.3389/fimmu.2023.1236806 (PMC10602784; doi:10.3389/fimmu.2023.1236806)

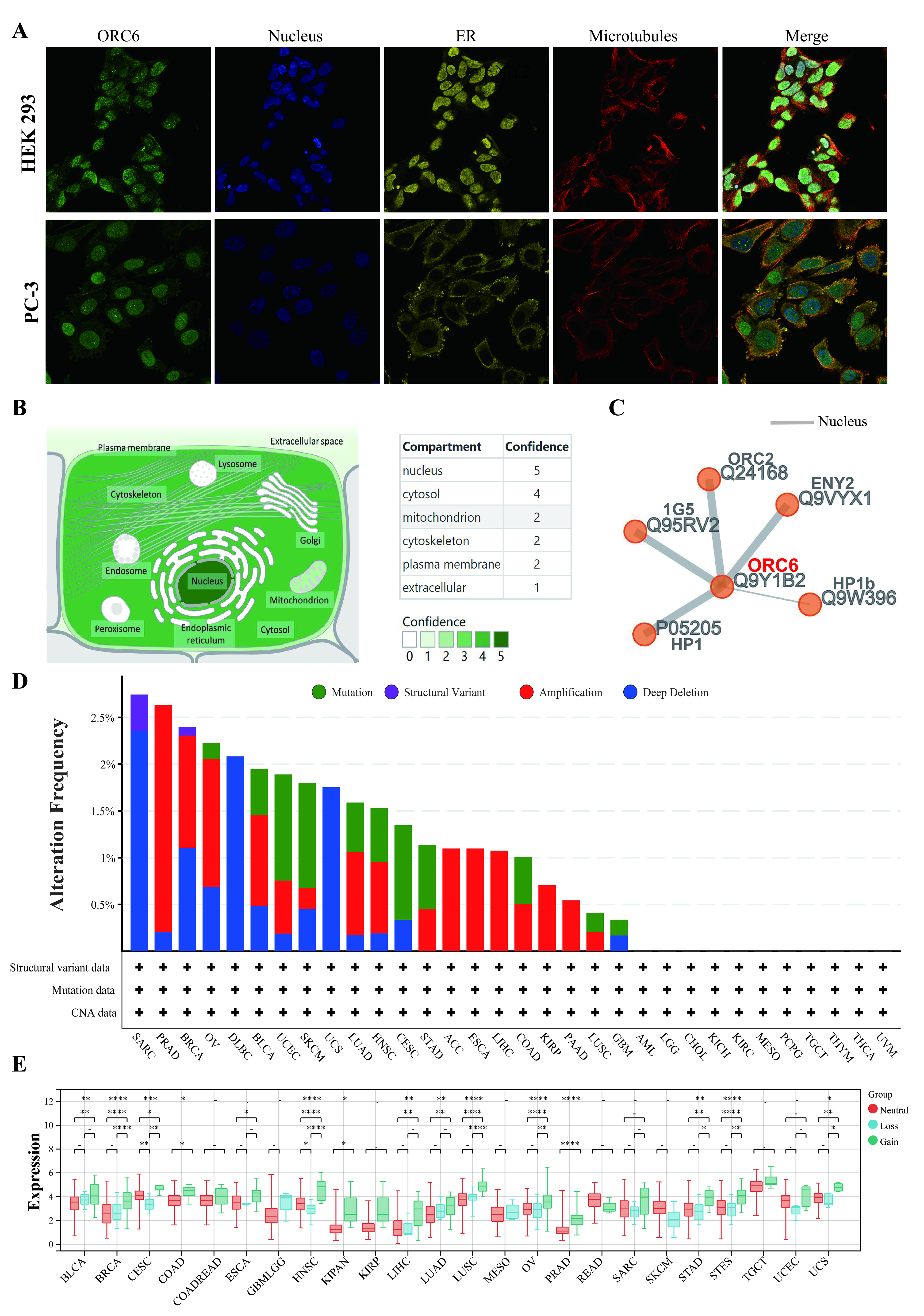

Supplement: Supplementary Figure S1 — ORC6 genetic alterations, localization, and interactions. (A) Immunofluorescence images and merged images of ORC6 protein, nucleus, endoplasmic reticulum (ER), and microtubules in HEK 293 and PC-3 cell lines. (B) ORC6 expression mapping was obtained through the Genecard website. (C) Protein-protein interaction (PPI) network presenting proteins that interact with ORC6. (D) Mutation types and frequencies of ORC6 in pan-cancer were obtained from the cBioPortal website. (E) The expression levels of ORC6 in various CNV status in pan-cancer. *p < 0.05, **p < 0.01, ***p < 0.001. [file Image_1.tif]

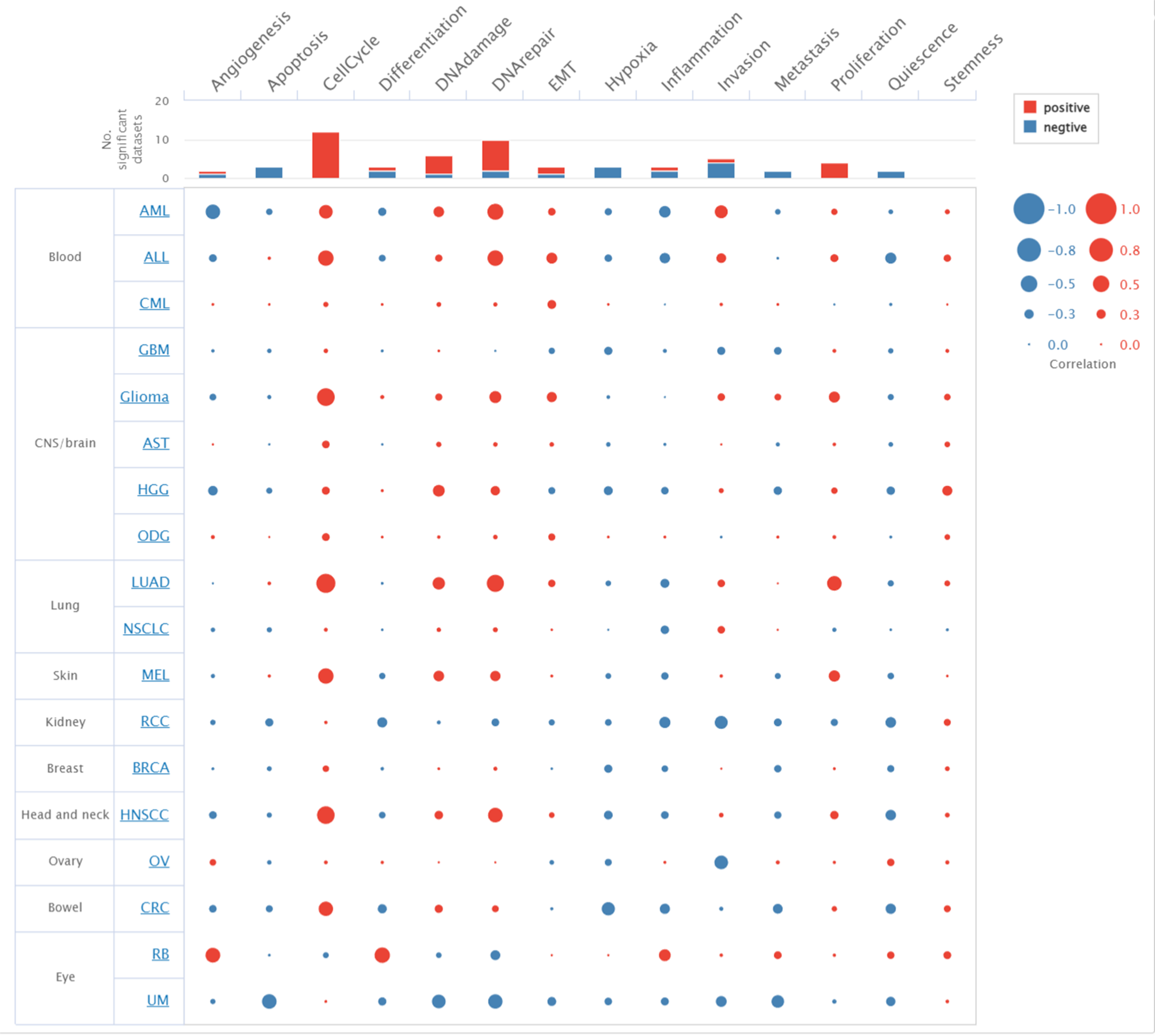

Supplement: Supplementary Figure S4 — Correlation between ORC6 expression and the functional status of 14 cancers. (A) The correlation between ORC6 expression and the functional status of 14 cancers was analyzed using single-cell sequence data from the CancerSEA database. [file Image_4.tif]
